# Framework for classifying compliance and medical immediacy among low-acuity presentations at an urban trauma center

**DOI:** 10.1186/s12245-015-0051-x

**Published:** 2015-03-19

**Authors:** Joshua G Behr, Rafael Diaz, Barry Knapp, Cynthia Kratzke

**Affiliations:** Virginia Modeling, Analysis and Simulation Center-VMASC, Old Dominion University, 1030 University Blvd, Suffolk, VA 23435 USA; Eastern Virginia Medical School, 700 West Olney Road, Norfolk, VA 23507 USA; New Mexico State University, 1780 East University Avenue, Las Cruces, NM 88003 USA

**Keywords:** Utilization, Compliance, Chronic condition, Financial, Emergent, Immediacy, Low acuity, Avoidable, Triage, Access, Emergency department

## Abstract

**Background:**

This research offers two exploratory frameworks, one for medical regimen compliance and one for medical immediacy. The first classifies compliance awareness, compliance mitigation, and financial limitation for those patients that exhibit nonadherence with a medical regimen. The second classifies medical immediacy and characterizes avoidable utilization.

**Methods:**

Representative sampling of adult patients presenting at an emergency department (62,000/ppy) triaged as low acuity; emergency department physician assessment of noncompliance with medical regimen for those patients with a complaint related to a chronic condition; and emergency department physician assessment of medical immediacy and avoidable utilization.

**Results:**

Physicians report 48.3% (95% confidence interval (CI) 43.5% to 53.1%) of patients with at least a single chronic condition are presenting with symptoms or complaint related to a chronic condition, and 39.6% (CI 31.7% to 47.4%) of these exhibit noncompliance with the medical regimen associated with that chronic condition. 16.4% (CI 6.6% to 26.1%) of the patients exhibit pseudo compliance, a belief that the medical regimen is in compliance when in fact it is not. If the patient had been in compliance, 85.9% (CI 77.0% to 94.8%) of the presenting conditions may have been mitigated. Noncompliance cases (34.5% (CI 22.0% to 47.1%)) are partly attributable to financial constraints. Further, 19.1% (CI 15.7% to 22.5%) are assessed as requiring no medical intervention and 3.4% (CI 1.8% to 4.9%) require immediate stabilization.

**Conclusions:**

A large portion of low-acuity presentations are related to a chronic condition and noncompliance with the associated medical regimen contributes to the need to seek medical services. Interventions addressing literacy and financial constraints may increase compliance and decrease utilization.

## Background

Emergency departments often treat low-acuity presentations. The current scope of responsibility positions the emergency department as a partner in the delivery of health care [1,2] and an important ‘adjunct’ to primary care [3] for underserved populations [4,5], those with health-care access barriers [6-11], and populations with mental illness and substance abuse [12-14]. Emergency departments function as a safety net for those with truncated primary and specialty care access [15-17], limited resources and transportation [18-20], and poor health literacy [21-23].

Emergency departments provide care within the context of both immediacy [24] and universality [25] to improve clinical outcomes, relieve pain and suffering, increase patient awareness and literacy, and, ultimately, promote quality of life and longevity. Emergency medicine and the emergency department grew out of a confluence of care systems, advances in equipment and procedures, and evolving societal attitudes about access to care [26]. The discipline of emergency medicine now encompasses issues, ‘related to timing, location, and access to care’ [27].

Failure to adhere to physicians’ advice is common [28,29]. Noncompliance with a medical regimen designed to assist in the management of a chronic condition may result in the need to seek the attention of a medical professional [30]. Nonadherence has been correlated with emergency department presentations [31,32], hospital admissions [33], increased health costs [34], and poor health outcomes [35-37]. The reasons for nonadherence with a regimen, especially a medication regimen, is recognized as complex [38-41] and includes, among many others, literacy or awareness barriers [42-47] and medication access barriers stemming from costs [48-51].

The focus of this research is upon two frameworks that have been developed to explore means of classifying low-acuity patient presentations. The first framework addresses patients with at least one chronic illness, who present with a chief complaint that is related to their chronic illness, and assesses these patients for various forms of non-adherence. The second framework addresses emergency department physician assessment of low-acuity utilization across a range of medical immediacy and the occurrence of avoidable utilization. The purpose of these frameworks is to understand motivations and root causes for low-acuity presentations; the study is not intended to indict a patient’s decision to seek services from the emergency department.

## Methods

### Patient population

The study population is derived from a representative randomized sampling (using sampling intervals) of adult patients (18+ years of age) presenting to a level 1 trauma urban hospital’s emergency department that sees approximately 62,000 patients annually. The catchment area for this regional urban hospital serves a diverse population including traditionally underserved and minority communities as well as uninsured and underinsured patient populations. The study population consists of low-acuity patients self-presenting or arriving via Emergency Medical Services (EMS) and triaged using the Emergency Severity Index (ESI) assigned either level 4 or level 5, a classification of patients based on urgency and resources needed [52,53]. A sampling of these triaged patients took place 24 h, 7 days a week over a period of approximately 8 weeks with 89% of approached subjects consenting to be part of the study. Since emergency department presentations are seasonal-cyclical in nature, using historical data we projected the expected volume of patient flow for both the day times and evening times. Using these historical data and estimated response rates, we established a sampling interval for each working shift within a 24-h period. As the study progressed, we periodically checked the evolving representativeness by cross-referencing both the financial and descriptive characteristics of the study population relative to the historical data. Post sampling, we contrasted this study sample with similar characteristics of the low acuity and overall ED populations (see Table 1). This research has been performed within the guidelines of the Helsinki Declaration. The study design and data gathering was monitored by the Institutional Review Board of Eastern Virginia Medical School.

**Table 1:** **Patient characteristics**

	**Sample low acuity**		**Population low acuity** ^**a**^ **(%)**	**Population overall** ^**b**^ **(%)**
	**No.**	**(%)**		
Sex				
Male	197	(39.2)	(43.6)	(41.7)
Female	306	(60.8)	(56.4)	(58.3)
Age (year)				
20 years or less (to 18)	61	(12.1)	(11.0)	(9.9)
21 to 30 years	185	(36.8)	(35.0)	(32.2)
31 to 40 years	98	(19.5)	(18.4)	(17.8)
41 to 50 years	92	(18.3)	(19.8)	(21.3)
51 to 60 years	47	(9.3)	(10.6)	(12.7)
61 or more years	20	(4.0)	(5.2)	(6.1)
Race/ethnicity				
White/Anglo/Caucasian	108	(21.5)	(24.1)	(26.2)
Black/African American	381	(75.7)	(73.0)	(70.5)
Hispanic/Latin American	6	(1.2)	n/a	n/a
Asian/Pacific Islander	6	(1.2)	n/a	n/a
Indian (India)	2	(0.4)	n/a	n/a

^a^Low acuity (triaged as 4 and 5) ED population during approximate sampling period estimated by patient characteristics. ^b^Overall (triaged as 1 through 5) ED population during approximate sampling period estimated by patient characteristics. n/a, not applicable.

### Patient interface

The prospective patient participant was informed of the nature and purpose of the study, potential benefits, and possible risks, and provided both verbal and written informed consent to a research associate, who was either a medical school or public heath student who underwent a training lattice for this study. During the process of assessment and treatment, an emergency department physician engaged in conversation with the patient to assess attributes of medical regimen compliance and medical immediacy and recorded these assessments using standardized instruments developed and piloted by the doctors. Information was recorded on project data sheets within the patient’s file and later imported into an electronic spreadsheet. Inter-coder reliability in data collection among the doctors was checked. These data are aggregated and presented in the two frameworks.

### Frameworks

#### Medical regimen compliance framework

Noncompliance has been characterized in the most general sense as, ‘the degree or extent of conformity to the recommendations about day-to-day treatment by the provider’ [54]. As such, compliance may include adherence to medication regimen as well as exercise and dietary guidelines. It is important to note that identifying a patient as exhibiting noncompliance is not intended to imply that the patient is responsible for lack of adherence or is resistant to following physician’s orders [34] as non-compliance often represents a reasoned decision on the part of the patient. The approach taken in this study is not to impeach the patient, rather, the intent is to contribute to the evolving understanding of the complexity of reasons patients are in noncompliance. This is accomplished by allowing physicians the opportunity to discuss with the patient and record in some systematic way explanations for the noncompliance.

In this study, the treating emergency department physicians documented both the presence of a chronic condition and the relationship of the chief complaint to the chronic condition. The physicians also assessed several factors (i.e., compliance awareness, compliance mitigation, and financial limitation) that may have conditioned the management of the chronic condition. To this end, the physicians reached consensus on wording and through discussion built a common understanding of definitions. Table 2 presents a summary of these attributes.

**Table 2:** **Medical regimen compliance**

**Compliance attribute**	**Conceptual definition**
Chronic condition	Patient has at least one chronic condition
Presenting condition related	The presenting condition is associated with a chronic condition
Noncompliance	The patient is not in compliance with his medical regimen
Cognizant non-compliance	The patient knows he is out of compliance with his medical regimen
Pseudo compliance	The patient believes he is in compliance, but, in fact, is not in compliance
Compliance mitigation	The presenting condition may have been mitigated if the patient had been in compliance
Financial limitation	Financial limitation is a factor in noncompliance

When assessing a patient, the physician registered the intensity of either agreement (i.e., strongly agree or agree) or disagreement (i.e., strongly disagree or disagree) with the conceptual definition of the attribute. This range, rather than a simple dichotomous (yes, no) response option, allowed us to code affirmative those patients that had relative compliance conformity. For example, over the course of a week, the missing of a single dose of medication for hypertension is technically noncompliance. However, the extent of the conformity suggests the patient is relatively compliant.

#### Medical immediacy framework

For the medical immediacy framework, the emergency department physicians collaborated in the development of a five-part ordered classification ranging from least to most immediate and a set of associated decision rules. Management of bias in individual interpretation of the language was addressed through training and the collaborative writing of the classification. Table 3 presents a summary of the decision rules developed to distinguish among the classification attributes.

**Table 3:** **Medical immediacy**

**Immediacy attribute**	**Immediacy decision rules**
Non-emergent/no medical intervention required	Patient has a non-emergent medical condition and this condition does not truly require the attention of a medical professional
Non-emergent/PCP visit prudent	Patient has a non-emergent medical condition that does not necessitate a visit to the emergency department. However, a visit to a primary care provider within several weeks may be prudent
Non-emergent/48-h window	Patient has a non-emergent medical condition that does not necessitate a visit to the emergency department. However, the patient ought to have medical attention within 48 h to prevent increased severity
Non-emergent/12-h	Patient has a non-emergent medical condition that does not necessitate a visit to the emergency department. However, the patient ought to have medical attention within 12 h to prevent increased severity
Emergent	Patient has an emergent medical condition requiring immediate stabilization

Although only patients triaged as low acuity participated in the study, during the process of treatment the physician again assessed medical immediacy using this classification. According to this approach, study patients potentially may be assessed by the treating emergency department physician to have an emergent condition and be in need of immediate stabilization. Such classification may have implications for the reliability of triage classifications [55]. One study notes that, ‘Although many ambulatory patients appear to have nonurgent conditions based on triage classification, a small but disturbing percentage of nonurgent patients are hospitalized’ [56].

In addition, it is acknowledged that the patient is the only one who can determine the need for presentation, although the treating physician determines what, if any, medical intervention may be needed. For example, the recording, ‘non-emergent/no medical intervention required’ is not intended to diminish the principle that it is the patient’s prerogative to seek medical attention and may indicate the patient could have managed the condition with OTC medication.

##### Avoidable presentations

Within this framework, the treating emergency department physician also documented an impression of the nature of the patient’s antecedent activity surrounding the chief complaint. This required engaging in conversation with the patient about the events and behaviors which may have given rise to the presenting condition. It is acknowledged that it is necessarily subjective to assess whether or not the condition could have been avoided and there may be bias depending on the physician’s background. To control for bias, the physicians met to build a common understanding and crafted the following statement:

*The patient*’*s presenting condition could be characterized by the prudent layperson as avoidable if the patient had recently altered his behavior or taken reasonable precaution.*

After conversing with the patient, the treating physician recorded either agreement or disagreement with this statement. The intent is not to assess the appropriateness of the emergency department presentation but rather to gain knowledge through a better understanding of the events and behaviors associated with the patient’s complaint [57-59]. The effort here is to judge, although imperfect, whether or not the condition could have been easily avoided.

Although there are various forms and language associated with prudent layperson laws among the several states that have adopted such provisions, the prudent layperson standard suggests that the determination of whether or not an emergency department visitation constitutes an emergency is based upon the patient’s perception of the immediacy of his/her presenting symptoms; if a person with average knowledge of health and medicine believes that the condition may result in impairment or dysfunction, or places his health in serious jeopardy, then there is an appropriate need to seek care. The physicians acknowledge that their clinical perspective and training makes them by definition non-lay yet attempted to view the context of the condition from the perspective of the ‘average person’.

## Results

### Medical regimen compliance framework

**Figure 1:**
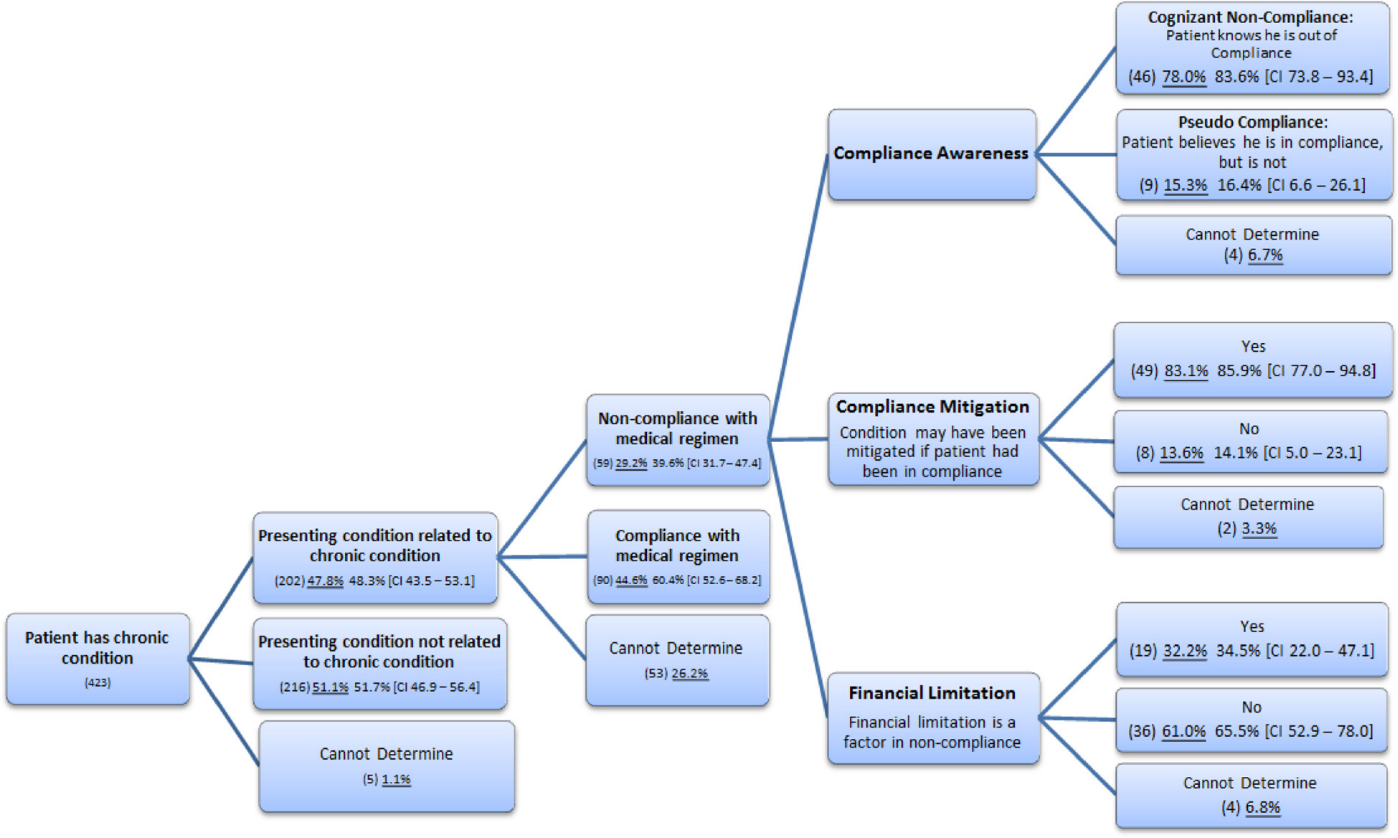
**Medical regimen compliance framework.** Note: underlined figures (percentages) are inclusive of the ‘Cannot Determine’ category.

The Medical Regimen Compliance Framework (Figure 1) illustrates the classification of presentations that are low acuity with a chief complaint related to a chronic condition. The treating emergency department physicians report that 48.3% (95% confidence interval (CI) 43.5% to 53.1%) of study patients with at least a single chronic condition are presenting with symptoms or complaint related to that chronic condition, and 39.6% (CI 31.7% to 47.4%) of these patients exhibit noncompliance with the medical regimen associated with that chronic condition.

#### Compliance awareness

The emergency department physicians assessed patient compliance awareness. Over 83% (CI 73.8% to 93.4%) of patients exhibit cognizance of their non-compliance. On the other hand, pseudo compliance is the concept that a patient knows that there is a medical regimen associated with his chronic condition and, on good faith, believes he is largely in compliance when in fact he is not. In other words, the patient is not openly aware of the nonadherence and believes that his activities satisfy the established medical regimen intended to manage the condition. Nearly 16.4% (CI 6.6% to 26.1%) of the patients believe they are managing their chronic conditions with the appropriate medical regimen but, in fact, remain noncompliant.

#### Compliance mitigation

The concept compliance mitigation gauges whether or not the nonadherence has contributed to the severity of the presenting condition. In 85.9% (CI 77.0% to 94.8%) of the noncompliant patients, the treating physicians determined that the presenting condition may have been mitigated if the patient had been in compliance.

#### Financial limitation

Financial limitation represents the cases in which personal financial constraints performed a contributory role in the nonadherence. Through eliciting conversations with the patient, the treating emergency department physician determined that in 34.5% (CI 22.0% to 47.1%) of the patients, nonadherence is partly attributable to the patient’s financial limitations.

### Medical immediacy framework

**Figure 2:**
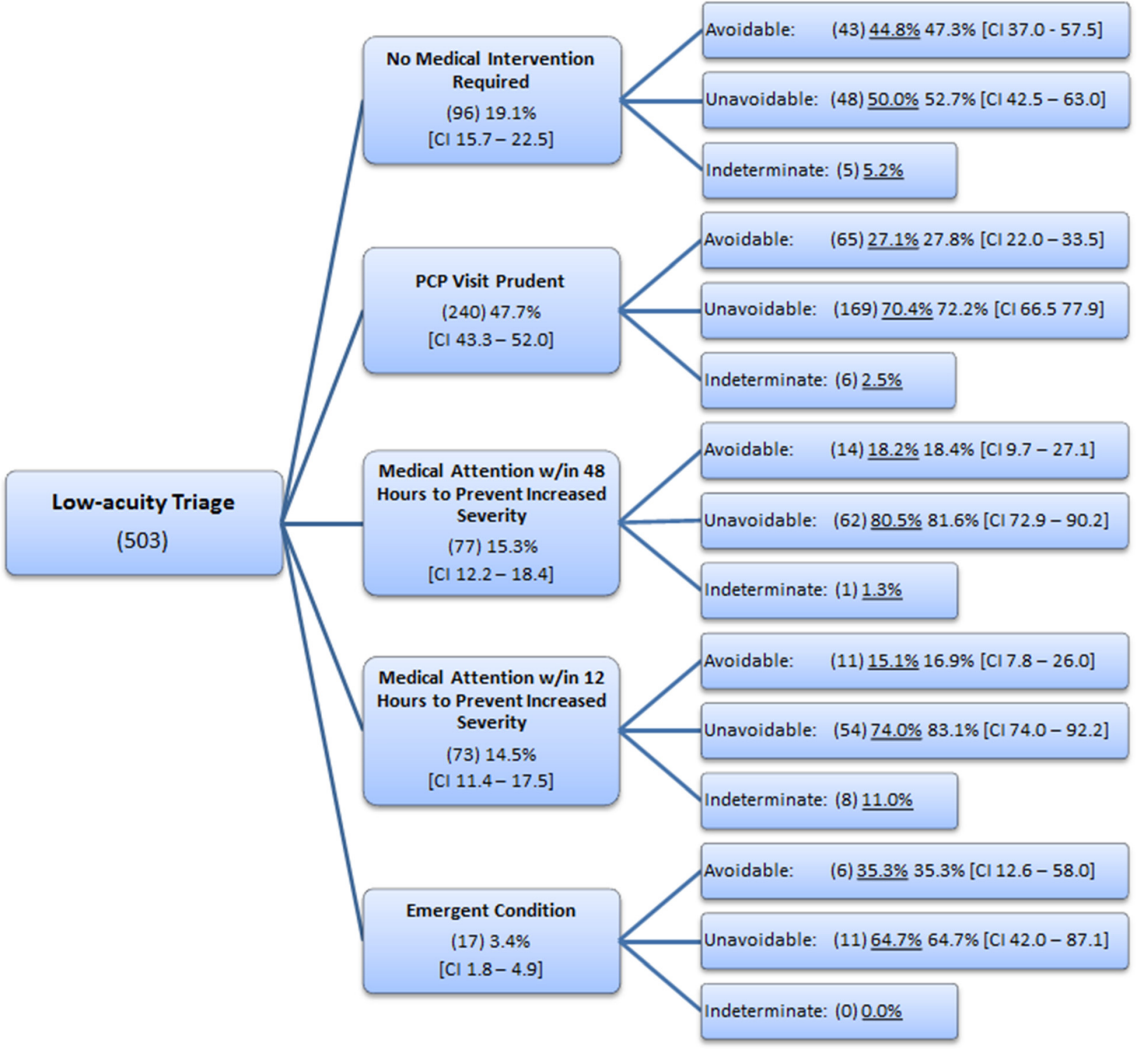
**Medical immediacy.**

The Medical Immediacy Framework (Figure 2) illustrates the disaggregation of low-acuity patients among the five-part ordered classification of medical immediacy and then offers the further separation of each classification into the utilization dichotomy (avoidable and unavoidable). Within this classification, 19.1% (CI 15.7% to 22.5%) are assessed by the emergency physicians as *No Medical Intervention Required*, 47.7% (CI 43.3% to 52.0%) *PCP Visit Prudent*, 15.3% (CI 12.2% to 18.4%) *Medical Attention within 48 Hours to Prevent Increased Severity*, and 14.5% (CI 11.4% to 17.5%) *Medical Attention within 12 Hours to Prevent Increased Severity*. The fifth category of medical immediacy, *Emergent Condition*, designates a medical condition that requires immediate stabilization. This category accounts for 3.4% (CI 1.8% to 4.9%) of physician assessed patients.

Patients falling within each classification are further disaggregated by the avoidable nature of their condition; that is, it was determined by the attending physician that if the patient had recently altered his behavior or taken reasonable precaution, then the presenting condition would have been avoidable. It is recognized that conceptually and operationally specifying a distinction between avoidable and unavoidable is difficult; deeming one condition unavoidable because it was an unforeseen accident and deeming another avoidable because the patient could have taken reasonable precaution are subjective. Nonetheless, the intent is to generate, although imprecise, a sense of the magnitude of avoidable conditions relative to unavoidable conditions that, informally, ‘*could have happened to nearly anybody and was an unavoidable accident*’. The percent of conditions that are unavoidable increases as the severity of each classification increases for the first four classifications (i.e., 52.7%, 72.2%, 81.6%, and 83.1%, respectively).

## Discussion

It may not be surprising that many low-acuity patients in the study had at least a single chronic condition and that nearly half (48.3%, CI 43.5% to 53.1%) have symptoms or a chief complaint related to that chronic condition. What is perhaps notable is the high rate of noncompliance (39.6%, CI 31.7% to 47.4%) with the associated medical regimen and the implications this may have for emergency department utilization; in a sizable majority of the cases (85.9%, CI 77.0% to 94.8%), had the patient been in compliance, the presenting condition may have been mitigated, and hence the patient’s decision to seek emergency department services less likely.

The utility of these exploratory frameworks may be found in identifying initial target populations that may be suitable for tailored interventions. For example, this research has observed the classification of roughly one-in-six noncompliant patients (16.4%, CI 6.6% to 26.1%) as exhibiting the characteristic of pseudo compliance. Interventions designed to address pseudo compliance must necessarily consider a range of issues including literacy relating to the management of the chronic condition [60-62], complicated and simultaneous medication schedules [63,64], better communication on behalf of the providers [65-67], and self-management [68]. However, for a clear majority of the noncompliant patients (83.6%, CI 73.8% to 93.4%), they are aware of their nonadherence. Interventions designed to address cognizant non-compliance must also consider a similar range of access, education, and communication factors. The physicians also documented that in roughly one third of the non-compliance situations (34.5%, CI 22.0% to 47.1%), a financial constraint played a role in the nonadherence.

In addition, this research asks the treating emergency department physician to assess the context of either the injury or the exacerbation in severity of the chronic condition. The point of interest is to encourage more in-depth discussion with the patient to gain knowledge of the context surrounding the condition and to explore, although imperfect, whether the presenting condition is unavoidable. Overall, 71.2% (CI 67.2% to 75.2%) of low-acuity triaged patients’ conditions truly were unavoidable in the sense that modest changes in behavior likely would not have avoided the development of the condition.

The classification scheme within the medical immediacy framework is able to distinguish between low-acuity presentations that require some form of primary care attention and those that do not require a medical intervention. These data show that nearly one-in-five (19.1%, CI 15.7% to 22.5%) patients triaged as low acuity do not require a medical intervention.

Another notable finding is the identification of patients whose conditions are triaged as low acuity, yet assessed by the treating emergency department physician as needing immediate stabilization (3.4%, CI 1.8% to 4.9%). Due to the emergent nature, these cases constitute appropriate utilization in the strictest sense. What is of interest, though, is the question, *What may account for the change in classification from the triaged low-acuity to the physician assessed emergent*? One possibility that may account for this disjuncture in assessments is that the severity of the patients’ conditions may have increased between the time of initial triage and subsequent assessment made at the time of treatment. Another possibility is that the triage protocol failed to adequately recognize or capture the truly emergent nature of the condition.

### Limitations

In this study, rigorous sampling methodology was deployed, trained technicians engaged in the recruitment and consent of participants, and physicians developed both the language and common understanding of the concepts embodied in the exploratory frameworks. Nonetheless, these activities were restricted to a single major urban trauma center and have a limited sample size; thus, confidence in the generalizability of the results must be appropriately tempered as caution ought to be used when extrapolating these findings to populations not addressed in this study. As with many studies, the results, while informative, pique interest in other yet-to-be answered questions.

Here, for example, the study is too limited to ascertain an explanation for the disjuncture between the low-acuity assessment at time of triage and latter physician assessment of emergent at time of treatment. What accounts for the disjuncture is difficult to ascertain, not because of the design of the framework but, rather, the relatively small sample size has not allowed for certainty in inferences. The framework in principle is able to solve this question. For example, given a large enough sample, if an examination of the ICD-9 diagnostic groups in which these anomalous patients fall evidences proportionate representation across all the groups, then this may suggest an increase in severity between triage and treatment. If, on the other hand, there is disproportionate representation of a particular grouping, then this may suggest that the particular condition may be more apt to be mis-triaged and, hence, the disjuncture is related more to a training issue rather than a clinical issue.

In addition, the study neither allows for the investigation of additional explanations for nonadherence nor analyzes expected change in utilization stemming from interventions. Further, neither does the study distinguish among chronic conditions. Finally, it is acknowledged that several of the concepts are necessarily inexact and that bias may be introduced stemming from the background of the treating physicians. These frameworks, though, are intended to be exploratory; these efforts may contribute to the rich literature documenting the complexity present in nonadherence and emergency department utilization.

## Conclusions

In summary, the use of these exploratory frameworks for medical regimen compliance associated with patients presenting with a chief complaint related to a chronic condition has allowed us to document the magnitude of nonadherence as well as several contributory factors to nonadherence (i.e., financial limitations and pseudo compliance). It also suggests the magnitude of presenting conditions that may have been mitigated if compliance had been maintained. Second, the exploratory framework for medical immediacy documents the magnitude of presentations that are triaged as non-emergent and later assessed as needing immediate stabilization by the treating physician as well as documenting the number of presentations that require no medical intervention. Together these frameworks illustrate the complexity in differences for whom and why patients with low-acuity conditions present at the emergency department and the conditions that give rise to nonadherence.
